# The Role of Neural Plasticity in Depression: From Hippocampus to Prefrontal Cortex

**DOI:** 10.1155/2017/6871089

**Published:** 2017-01-26

**Authors:** Wei Liu, Tongtong Ge, Yashu Leng, Zhenxiang Pan, Jie Fan, Wei Yang, Ranji Cui

**Affiliations:** ^1^Jilin Provincial Key Laboratory on Molecular and Chemical Genetic, The Second Hospital of Jilin University, 218 Ziqiang Street, Changchun 130041, China; ^2^Anesthesiology Department, The Second Hospital of Jilin University, 218 Ziqiang Street, Changchun 130041, China; ^3^Anesthesiology Department, The Third Hospital of Jilin University, 126 Xiantai Street, Changchun 130033, China

## Abstract

Neural plasticity, a fundamental mechanism of neuronal adaptation, is disrupted in depression. The changes in neural plasticity induced by stress and other negative stimuli play a significant role in the onset and development of depression. Antidepressant treatments have also been found to exert their antidepressant effects through regulatory effects on neural plasticity. However, the detailed mechanisms of neural plasticity in depression still remain unclear. Therefore, in this review, we summarize the recent literature to elaborate the possible mechanistic role of neural plasticity in depression. Taken together, these findings may pave the way for future progress in neural plasticity studies.

## 1. Introduction

The establishment and realization of neural functions are based on generation, transformation, and storage of information in neural networks. The brain is developing and progressing at high speed in the six- to nineteen-year-old age group, and the unique plasticity of neural development is crucial to mature neural function. In a neural network, neurons are the fundamental functional units that integrate and transmit signals in response to intrinsic and extrinsic information [1]. Neuronal functions are dynamic processes that occur in response to environmental stimuli, emotions, injury, and so forth. This is the theoretical basis of neural plasticity, which is an umbrella term to describe structural and functional changes in the brain in response to various stimuli, including stress and depression. Depression is a prevalent, chronic, and recurrent disease. Depression, one of most devastating diseases, has a worldwide lifetime prevalence of 20%. Moreover, to patients with depression, depression not only brings profound mental agony but also causes pathophysiological disorders and enhances susceptibility to some diseases, for instance, cardiac diseases and cerebrovascular illness [2]. Therefore, patients with depression suffer from higher mortality than the healthy population. Unfortunately, to date, no completely effective treatments for depressed patients have been developed. Currently available antidepressant treatments, whether medications, psychotherapies, or other methods, have limited efficacy in depression and can cause significant side effects [2]. Hence, it is profoundly significant to explore the pathophysiology of depression. Though a large number of studies on the correlation between depression and neural plasticity have revealed some of their mechanisms, the neurobiological mechanisms of depression are still not well known. Negative stimuli, such as stress, pain, and cognitive impairment, can result in both depression and changes in neural plasticity. The neuroplasticity hypothesis of major depressive disorder proposes the theory that dysfunction of neural plasticity is a basic pathomechanism of the disorder [3]. However, depression is not an inexorable outcome of dysfunction of neural plasticity. To our knowledge, there are no authoritative research results or expert consensus to confirm whether depression or changes in neural plasticity are the initial factor. Most of the studies suggest that depression and dysfunction of neural plasticity act on and influence each other. In this perspective, we review the recent literature to elaborate what is known about neural plasticity in depression to pave the way for ongoing and future studies.

## 2. Hippocampal Plasticity in Depression 

The hippocampus is the most commonly studied brain region in depression research. From a structural point of view, the hippocampus is part of the limbic system and develops nerve fiber connectivity with emotion-related brain regions, for instance, the prefrontal cortex and amygdala. In addition, the hippocampus contains high levels of glucocorticoid receptors and glutamate and regulates the hypothalamus-pituitary-adrenal (HPA) axis, which makes it more susceptible to stress and depression. Changes in hippocampal plasticity can result from stress and other negative stimuli. Stress impacts hippocampal plasticity in many ways. Chronic and severe stress has been shown to impair hippocampus-dependent explicit memory in animal models of depression [4]. This effect can be explained by changes in hippocampal synaptic plasticity modeled by long-term potentiation (LTP) and long-term depression (LTD). Hippocampal synaptic plasticity is widely considered to play an important role in hippocampus-dependent explicit memory formation [5]. Severe stress can impair LTP and enhance LTD in the hippocampi of rodent models [6, 7]. Stress can also decrease neuronal dendrite branching and plasticity in the hippocampus [8]. In addition, stress can trigger activation of the hypothalamic-pituitary-adrenal axis, increase level of corticosteroids, and downregulate hippocampal neurogenesis [9]. Cognitive impairment can enhance long-term potentiation in the CA1 region and markedly elevate protein levels of the *α*-amino-3-hydroxy-5-methyl-4-isoxazolepropionic acid (AMPA) receptor subunit GluA1 in the mouse hippocampus and then induce depression in mouse models [10]. In addition, neuropathic pain-induced depressive-like behavior may be associated with hippocampal neurogenesis and plasticity through tumor necrosis factor receptor 1 signaling [11]. Hippocampal plasticity in depression involves hippocampal volumetric changes, hippocampal neurogenesis, and apoptosis of hippocampal neurons.

### 2.1. Synaptic Plasticity in the Hippocampus

Synaptic plasticity is one of the most fundamental and important functions of the brain. The efficacy of transmission at a synapse depends on modulation of the connectivity between neurons and neuronal circuits during adaptation to the environment [12]. Stress has profound effects on synaptic plasticity in the hippocampus and presents different influences in different subfields of the hippocampus. Stress can impair LTP in CA3 while facilitating LTD and spike-timing-dependent LTD (tLTD) in CA1 [12]. In addition, depression can downregulate synaptic proteins and growth factors required for hippocampal LTP in animal models.

Electroacupuncture can alleviate depression-like behaviors and reverse the impairment induced by long-term potentiation in the CA1 synapses of the hippocampus in depressive rats [13]. Physical exercise may prevent changes in synaptic plasticity and increases in synaptic transmission in hippocampal CA1 pyramidal neurons caused by stress but cannot reverse the present glutamatergic synaptic alterations induced by depression [14]. Glucocorticoid receptor antagonists and monoaminergic antidepressants can protect against negative synaptic plasticity in CA1 induced by stress. TJZL184, a monoacylglycerol lipase inhibitor, exhibited antidepressant effects by enhancing adult neurogenesis and long-term synaptic plasticity in the dentate gyrus of the hippocampus [15].* Lycium barbarum* was found to reduce depression-like behavior mediated by enhanced synaptic plasticity in the hippocampus of rats [16].

### 2.2. Hippocampal Volumetric Changes in Depression

It has been widely reported that there is a significant reduction in hippocampal volume in depression patients [17]. This situation was found in both adult and adolescent depressed patients, whether they were in their first or recurrent depressive episodes. A recent study reported that, in female patients with recurrent familial pure depressive disorder (rFPDD), volumetric reductions of the right hippocampal body and tail were significantly larger than those of the left, while the whole brain volume was approximately equal to that of healthy subjects [18]. Consistent with this, a significant increase in right hemispheric hippocampal gray matter volume has been found in elderly patients with severe depression treated with electroconvulsive therapy [19, 20]. However, hippocampal volumetric reduction was also found in patients who had recovered from depression [17]. The volumetric changes may result from a neurodegenerative reaction to increased glucocorticoid levels in depression [20]. The changes in synaptic plasticity induced by depression are associated with structural and functional changes in the hippocampus. The volume reduction of the prefrontal cortex and hippocampus may also result from the disruption and atrophy of neurons and glia in depression [21, 22]. Nonetheless, the hippocampal volumetric changes are not associated with the severity of depression [18]. Evidence supports that larger hippocampal volumes indicate quicker recovery in depressed individuals [23]. This can be explained by hippocampal regulation in stress reactivity. Reduced hippocampal volumes may be a neural scar marker of depression and a vulnerability marker for future episodes [17]. The clinical application of hippocampal volumetric changes still needs large-sample research to confirm.

### 2.3. Hippocampal Neurogenesis

Brain neurogenesis lasts from birth to adulthood in many animals, including humans. Hippocampal neurogenesis occurs markedly in the dentate gyrus, with approximately 700 granule cells born daily, corresponding to an annual turnover of 1.75% of the neurons within the renewing fraction [24].

The rate of hippocampal neurogenesis decreases modestly with age. Compared with the millions of granule cells in the granular layer of the hippocampus, newborn neurons are few in number, but they can be sufficient to achieve functional significance [25]. Though the rates of neuronal regeneration are comparable in middle-aged humans and mice, the patterns of adult hippocampal neurogenesis are significantly different between them. In humans, approximately one-third of hippocampal neurons are subject to exchange. By contrast, the proportion is 10% in mice [26]. The relative decline rate of hippocampal neurogenesis during adulthood in humans is lower than that in mice. In addition, hippocampal neurogenesis in mice is additive, and newborn neurons can compensate for lost cells, while new neurons in humans cannot keep up with the losses [25]. Therefore, hippocampal neurogenesis in humans may have an additive function in the circuitry and enhance synaptic plasticity to achieve maximum impact.

The neurogenic hypothesis of depression emphasizes the theory that impaired adult hippocampal neurogenesis results in depression, and newborn neurons in the adult brain are critical to mood regulation and antidepressant efficacy [27, 28]. Impaired adult hippocampal neurogenesis and depression may be reciprocally causative [29]. High levels of glucocorticoids in depression also hinder adult hippocampal neurogenesis, but adrenalectomy can promote adult hippocampal neurogenesis.

The effects of antidepressant treatments on adult hippocampal neurogenesis have shown discrepancies in different species. In rodents, most antidepressant treatments that are used in humans, including electroconvulsive shock and medication, were subsequently shown to facilitate hippocampal neurogenesis [30–34]. However, the facilitation of fluoxetine treatment was sensitive to stress, corticosterone levels, and route of medication [29]. The effects of fluoxetine treatment on non-human primates are similar to those in rodents. In addition, electroconvulsive shock can also boost hippocampal neurogenesis in non-human primates [35]. There is a lack of research data to illustrate the effects of medication on non-human primates. In humans, total dentate granule cell number and dentate gyrus size in medicated patients with depression are larger than those in nonmedicated patients based on postmortem studies [36]. Selective serotonin reuptake inhibitors, lithium treatment, and electroconvulsive shock produce larger increases in hippocampus volume in treated depressed patients than in nontreated patients [37, 38]. In line with this observation, research evidence from the hippocampal subfields has revealed larger dentate gyri in medicated depressed patients [39]. These data are relevant to multiple forms of neural plasticity and suggest an increase in hippocampal neurogenesis. Nevertheless, there is no adequate evidence to establish that hippocampal neurogenesis is necessary for antidepressant efficacy, and its increase is sufficient for antidepressive therapy.

### 2.4. Hippocampal Apoptosis in Depression

Proliferation, differentiation, and apoptosis are continuous progressions in adult hippocampal neurons. Many studies have demonstrated that depression and stress can induce hippocampal apoptosis in rodents, non-human mammals, and humans, though hippocampal apoptosis can also be found in nondepressed rodents [40]. Similarly, hippocampal apoptosis may result in depression. Evidence showed an increase of apoptosis in dentate gyrus of maternal rats with repeated separation of their pups and impairment of memory capability with depression-like behavioral changes [41]. In maternal-separation rat models, tadalafil, a phosphodiesterase type 5 inhibitor, exerts antidepressant effects by suppressing maternal-separation-induced apoptosis and increasing cell proliferation in the dentate gyrus [42]. Though some studies support the idea that hippocampal apoptosis is a causative factor in hippocampal volumetric changes, histopathological studies on depressed patients have yielded inconsistent results [43]. There are differences in the stimulative effects of chronic depression and acute depression on hippocampal apoptosis. In animal models and human studies, chronic depression showed longer lasting apoptosis-promoting effects in the hippocampus than acute depression [40]. The apoptosis-promoting effects induced by acute depression can fully subside in one day of recovery, while the adverse effects in chronic depression may need up to three weeks for recovery. However, it is uncertain in what stage depression and stress start to mediate apoptosis progression. In addition, their effects showed differences among subfields of the hippocampus. Compared with the apoptosis increase in the whole dentate gyrus caused by acute depression, the number of cells in the granular cell layer can increase even as the cell count in the whole dentate gyrus is declining [44]. This discrepancy may be due to the different sensitivities of granular cells to acute and chronic stress.

In addition to tadalafil (mentioned above), several types of drugs may have antidepressant effects owing to hippocampal apoptosis. For instance, venlafaxine, a serotonin/norepinephrine dual reuptake inhibitor, suppresses hippocampal apoptosis by upregulating brain-derived neurotrophic factor [45]. In addition, fluoxetine, a 5-hydroxytryptamine reuptake inhibitor, regulates hippocampal plasticity by alleviating the upregulation of synaptosomal polysialic neural cell adhesion molecule caused by depression and elicits an antiapoptotic response in the hippocampus [46].

## 3. The Prefrontal Cortex in Depression

The prefrontal cortex (PFC), as a significant nerve center of thinking and behavior regulation in the brain, is also associated with depression [47]. In view of anatomical connectivity and functional specialization, the prefrontal cortex is divided into two subregions: ventromedial prefrontal cortex (vmPFC) and dorsolateral sectors (dlPFC) [48]. VmPFC involves the regulation of affection, including the generation of negative emotion, and dlPFC mediates cognitive functions, such as intention formation, goal-directed action, and attentional control [49]. The two sectors have both been shown to have significant roles in depression. However, their effects present discrepancies, according to reports in the literature. Functional imaging studies have shown opposite changes of activity in the two sectors: during the progression of depression, hyperactivity appeared in the vmPFC, while hypoactivity appeared in dlPFC; in the recovery phase in response to psychotherapy or medication for depression, hypoactivity was found in the vmPFC, while hyperactivity was found in dlPFC [48, 50–53]. Furthermore, in lesion models, dlPFC loss can aggravate depression, whereas vmPFC loss can exhibit an alleviative effect on depression [54, 55]. Dysfunction caused by dlPFC damage in stroke is considered a predisposing factor to poststroke depression [56]. In addition, a decrease of cortical thickness in the right vmPFC, which occurs in the early stages of neurodevelopment, results in depression in preschoolers [57]. Volume reduction of the prefrontal cortex may result from the disruption and atrophy of neurons and glia in depression, as observed in the hippocampus [21, 22]. Energy and glutathione metabolic pathways in the prefrontal cortex were shown to be significant biological pathways in depressive rats [58]. Many studies have indicated that changes in glutamate metabolism were associated with depression [59–67]. In a stress-induced depressive mouse model, the prefrontal cortex in depression showed a significant reduction of glutamate in the GABAergic pathway, which may contribute to depression [62]. Activation of metabotropic glutamate receptor 3, which plays a significant role in regulating the function and cognition of the prefrontal cortex, can result in long-term depression in the medial prefrontal cortex of rats in vitro [68]. The GRIN2A gene, which encodes the glutamatergic N-methyl-D-aspartate (NMDA) receptor subunit epsilon-1 in the prefrontal cortex, is probably disturbed in the regulation of synaptic plasticity in depression [69].

In addition, a novel miRNA (miR-101b) was found to be downregulated in depression and could decrease mRNA and protein levels of glutamate transporter SLC1A1 in the prefrontal cortex [59]. In addition, in the medial prefrontal cortices of chronic unpredictable mild stress-induced depressive mice, there was a downregulation of mRNAs encoding proteins for the GABAergic synapses, dopaminergic synapses, synaptic vesicle cycle, and neuronal growth and an upregulation of miRNAs of regulating these mRNAs [70]. In a chronic corticosterone-mediated depressive rat model, the majority of the related miRNAs and associated gene networks showed glucocorticoid receptor element binding sites; this is a potential mechanism whereby corticosterone may mediate depression [71].

There is also a decrease in prefrontal hemodynamic responses in depression and a significant and positive correlation between prefrontal hemodynamic responses and the role of the emotional domain [72, 73]. In addition, the lack of activation of oxygenated hemoglobin in the prefrontal cortex indicates that it may be a mechanism of depression [74]. Observing changes in hemoglobin concentration in the prefrontal cortex detected by near-infrared spectroscopy may be a convenient approach to evaluate and predict antidepressant improvement in late-onset depression [58]. Furthermore, increases in mean oxygenated hemoglobin may be positively correlated with the severity of depression [75].

Repetitive transcranial magnetic stimulation of the dorsomedial prefrontal cortex (dmPFC) and dlPFC exhibits effectiveness and safety in treatment-resistant depression [76–80]. In electroconvulsive therapy for depression, an early decrease of intralimbic functional connectivity and a later increase of limbic-prefrontal functional connectivity were found [81]. Epidural prefrontal cortical stimulation over the PFC has also been shown to be a promising novel therapeutic method for treatment-resistant depression [82]. Positive emotional learning can facilitate N-methyl-D-aspartate (NMDA) receptor-dependent synaptic plasticity in the medial prefrontal cortex and then exert positive effects on promoting rehabilitation in depressive rats [83]. Some studies have reported that NMDA receptor antagonists, such as ketamine and lanicemine, can increase mammalian target of rapamycin complex 1 (mTORC1) signaling by activating threonine kinase (AKT) and extracellular signal-regulated kinase (ERK) signaling pathways and increase synaptic number and function in the prefrontal cortex [2, 84, 85]. A recent study on protein level changes in the prefrontal cortex suggested that treatment with the tricyclic antidepressant clomipramine in neonates was a reliable model to study the effects of antidepressants on the early phase of brain development [86]. Hence, the effects of antidepressant treatment on early brain development may induce constant pathological changes in the prefrontal cortex. YY-23, a new extractive compound, and fluoxetine can reverse the inhibitory effects of chronic mild stress on spontaneous burst firing of medial prefrontal cortex pyramidal neurons in depression [87]. Mecamylamine, a nicotinic antagonist, is a novel antidepressant that exerts antidepressant actions by increasing PFC levels of BDNF and monoamines [88]. Interestingly, in a depressive rat model, nutritional supplements, such as n-3 polyunsaturated fatty acids (PUFA), may prevent the development of depression by impeding HPA axis hyperactivity [89]. This study suggests that dystrophy may be another mechanism of depression.

## 4. Amygdalar Changes in Depression

The amygdala plays a significant role in affective modulation and memory encoding [90]. The amygdala is also a critical site of neuronal plasticity for fear conditioning [91]. Morphological and functional changes of the amygdala associated with depression have been verified in many studies [92, 93]. In contrast, with the hippocampus and prefrontal cortex, stress and depression enhance synaptic plasticity in the amygdala and the ventral emotional network [3]. Stress was found to induce dendrite retraction in the PFC and hippocampus, while it induced dendritic arborization of pyramidal and spiny neurons in the basolateral amygdala [12]. Expression of brain-derived neurotrophic factor (BDNF), which is known to play a central role in synaptic plasticity induced by stress, increased in the basolateral amygdala but decreased in the hippocampal CA3 in rats [9, 94]. Depression disrupted glutamate signaling at the NMDA receptor in the amygdala in humans [95]. Neonatal glucocorticoid treatment enhanced LTP response and the phosphorylation level of MAPK in the lateral nucleus of the amygdala and promoted depression-like behavior in adult rats [91].

Amygdala kindling, as a classic model of temporal lobe epilepsy with convulsion, can cause depression-like behaviors in both immature rats and adult rats [96]. Amygdalar functional connectivity differs in late-life depression phenotypes, and this discrepancy may be a criterion to distinguish phenotypes of late-life depression and evaluate the severity [97].

In addition, the volume of the amygdala varied with the severity of the depression [98]. Interestingly, a recent study showed that larger gray matter volume in the bilateral amygdala was found in first-degree relatives of depressed patients [99]. Furthermore, amygdala perturbations caused by negative stimuli, which elicit greater amygdala activation, might be an early and subtle risk marker for depression [100]. Recent evidence suggests that postpartum depression can increase amygdalar response to infant stimuli and decrease bilateral amygdala-right insular cortex connectivity [101]. The latter may have a stimulative effect on depression and anxiety. However, abnormal functional connectivity in depression is discrepant in the left amygdala [102]. In the left amygdala, the functional connectivity decreased in the amygdala positive network, while it increased in the amygdala negative network. In a clinical study of early-childhood-onset depression, functional connectivity was reduced in the bilateral amygdala [103]. Abnormal amygdala functional connectivity is also found in late-onset depressed patients [104]. Hence, a distributed neuronal network including cortical and limbic regions rather than a discrete brain region contributes to depression. The amygdala-associated frontolimbic circuits, amygdala-dorsal lateral prefrontal cortex, and amygdala-ventromedial prefrontal cortex, which integrate affective processes, may have characteristic dysfunctions in adolescent depression [104]. These circuits may change exponentially in association with depression severity and potentially be considered as a biomarker to analyze the effect of treatment on depression. Interestingly, some of the amygdalar changes in depression differ by gender. A recent study indicated that women but not men possess an IL18 haplotype that increases threat-related left centromedial amygdala reactivity and boosts susceptibility to stress-related depression by promoting proinflammatory responses [105]. Depression-associated single-nucleotide polymorphisms can regulate the expression of the bicaudal C homolog 1 (BICC1) gene and decrease its promoter activity on the PKA signaling pathway in amygdalar neurons [106]. These changes may cause mood disorders. In addition, prenatal maternal depression can influence the functional connectivity of the amygdala in early postnatal life, particularly in 6-month-old infants [107]. Prenatal maternal depression can also incur the risk of aggression in offspring [108]. In contrast, many studies have suggested that amygdala hyperactivity may improve symptoms of depression [109].

Some antidepressant treatments have been shown to play a role in amygdala regulation. Transcutaneous vagus nerve stimulation is a noninvasive peripheral neuromodulation therapy administered at the ear for depressed patients and has been shown to be effective for depression treatment [110]. It can promote amygdala-lateral prefrontal network resting state functional connectivity in the right amygdala of depressed patients [111]. Real-time fMRI neurofeedback training is another novel noninvasive treatment for depression [112]. It can enhance blood-oxygenation-level-dependent activity in the amygdala and benefit depressed patients. In addition, the effects of electroconvulsive therapy in patients with depression may also be associated with neuroplasticity changes in the amygdala, and this phenomenon may be due to neurotrophic processes, including neurogenesis [112]. Medication associated with the amygdala in depressed patients includes quetiapine, citalopram, and ketamine [113–115]. In depressive rat models, the amygdala has shown a significant role in fluoxetine-stimulated cell survival and a potential to modulate antidepressant action in hippocampal neurogenesis [116].

## 5. Neural Plasticity in Other Brain Regions in Depression 

The ventral striatum participates in the mechanisms of natural reward, and its dysregulation contributes to symptoms of anhedonia in depression [4]. Chronic stress can cause long-term adaptations in the ventral tegmental area-accumbens pathway that may contribute to its dysregulation in major depression [4].   *α*_1_-Adrenoceptor dependent downregulation of the membrane GluR1 subunit in the mouse ventral tegmental area mediated the depressive-like behavior induced by lipopolysaccharide [117]. In rats with postpartum depression, gestational stress could decrease dendritic length, branching, and spine density on medium spiny neurons in the nucleus accumbens shell and promote depressive-like behavior in the early/mid-postpartum phase [118].

Hypothalamic synaptic plasticity in depression can be caused by increased mRNA expression of synaptotagmin I and synapsin I, and the latter may contribute to depression-like behaviors and HPA axis hyperactivity [119]. In addition, the extracellular matrix may be involved in synaptic stabilization and transmission and may modulate synaptic plasticity in the central nervous system [120]. In recent studies, modeling of bidirectional modulations in synaptic plasticity, designed to reveal the mechanism of long-term potentiation and long-term depression, suggested that Ca^2+^/calmodulin (CaM) pool size played a critical role in coordinating LTP/LTD expression [121].

## 6. Summary and Conclusion

Overall, neural plasticity is a vital feature of the brain in response to intrinsic and extrinsic stimuli, including stress and depression. Mounting clinical and basic research studies have illuminated the correlations between neural plasticity and depression. As the summaries in Tables 1 and 2, the effects of depression on neural plasticity are complex pathophysiological processes, involving multiple encephalic regions, such as the hippocampus, prefrontal cortex, and amygdala as well as complicated interactions of many signal pathways, such as NMDA, glutamate, and glucocorticoid. On the other hand, the changes in neural plasticity induced by stress and other negative stimuli can contribute to the onset and development of depression. The majority of antidepressant treatments, including psychotherapies, physiotherapies, and medications, exert antidepressant effects associated with neural plasticity. Unfortunately, to date, no ideal and completely effective treatment has been found for depressed patients. Though we have done extensive work in this review, the detailed mechanisms of neural plasticity in depression still remain unclear. Targeting neural plasticity in depression may lead to novel breakthroughs.

## Figures and Tables

**Table 1:** Changes of neural plasticity induced by depression in various brain regions.

Brain region	Changes of neural plasticity	Mechanisms
Hippocampus	Synaptic plasticity	(1) Impairment of LTP in CA3
		(2) Facilitation of LTD and tLTD in CA1
		(3) Downregulation of synaptic proteins and growth factors
	Volumetric changes	(1) Disruption and atrophy of neurons and glia
		(2) Neurodegenerative reaction to high levels of glucocorticoid
	Neurogenesis	(1) Hindered by high levels of glucocorticoids and enhanced by adrenalectomy
		(2) Additive effects in mice, while reduced in humans
		(3) Additive function in the circuitry
	Apoptosis	(1) Depression promotes apoptosis in the hippocampus
		(2) The effects caused by chronic depression last longer than those of acute depression

Prefrontal cortex	Synaptic plasticity	(1) Disturb expression of NMDA receptor gene
		(2) Downregulation of proteins for the GABAergic synapses, dopaminergic synapses, synaptic vesicle cycle
		(3) Downregulation of mRNA and protein levels of glutamate transporter SLC1A1
	Activity in vmPFC and dlPFC	(1) Hyperactivity in vmPFC and hypoactivity in dlPFC during progression of depression; hyperactivity in dlPFC and hypoactivity in vmPFC during recovery phase
		(2) Decrease of cortical thickness of right vmPFC through disruption and atrophy of neurons and glia
	Energetic metabolism	(1) Reduction of glutamate in the GABAergic pathway
		(2) Activation of metabotropic glutamate receptor 3
		(3) Disturbed expression of NMDA receptor gene
		(4) Downregulation mRNA and protein levels of glutamate transporter SLC1A1
	Hemodynamic responses	(1) Lack of activation of oxygenated hemoglobin
		(2) Changes in hemoglobin concentration may be positively correlated with severity of depression

Amygdala	Synaptic plasticity	(1) Increased expression of BDNF
		(2) Disrupted glutamate signaling at the NMDA receptor
		(3) Neonatal glucocorticoid treatment enhances LTP response
	Volumetric changes	(1) Larger gray matter volume in the bilateral amygdala
	Functional connectivity	(1) Decreased bilateral amygdala-right insular cortex connectivity
		(2) In the left amygdala, the functional connectivity decreased in positive network and increased in negative network
		(3) Amygdala-associated brain circuits may change with depression severity
		(4) Prenatal maternal depression increases functional connectivity in infants

Ventral striatum		(1) Caused long-term adaptations in the ventral tegmental area-accumbens pathway
		(2) *α*_1_-Adrenoceptor dependent downregulation of the membrane GluR1 subunit
		(3) Decreased dendritic length, branching, spine density on medium spiny neurons in the nucleus accumbens shell

Hypothalamus	Synaptic plasticity	(1) Increased mRNA expression of synaptotagmin I and synapsin I

**Table 2:** Neural plasticity in the treatment of depression.

Therapy	Model	Mechanism and influence of neural plasticity
Electroacupuncture	Rats	Reverses the impairment induced by long-term potentiation in CA1 synapses of hippocampus

Electroconvulsive shock	Rats and humans	Facilitates hippocampal neurogenesis, an early decrease of intralimbic functional connectivity and a later increase of limbic-prefrontal functional connectivity, and makes neuroplasticity changes in the amygdala due to neurotrophic processes including neurogenesis

Transcutaneous vagus nerve stimulation	Humans	Promotes amygdala-lateral prefrontal network resting state functional connectivity in right amygdala

Real-time fMRI neurofeedback training	Humans	Enhances blood-oxygenation-level-dependent activity in amygdala

Positive emotional learning	Rats	Facilitates N-methyl-D-aspartate (NMDA) receptor-dependent synaptic plasticity learning

Physical exercise	Rats	Prevents changes in synaptic plasticity and increases in synaptic transmission in hippocampal CA1 pyramidal neurons caused by stress

Nutritional substance supplementation	Rats	Prevents the development of depression through impeding HPA axis hyperactivity

Glucocorticoid receptor antagonists	Rats	Protects against negative synaptic plasticity in CA1 induced by stress

Monoaminergic antidepressants	Rats	Protects against negative synaptic plasticity in CA1 induced by stress

TJZL184 (a monoacylglycerol lipase inhibitor)	Rats	Enhances adult neurogenesis and long-term synaptic plasticity in the DG of the hippocampus

*Lycium barbarum*	Rats	Enhances synaptic plasticity in the hippocampus

Lithium (selective serotonin reuptake inhibitor)	Rats and humans	Facilitates hippocampal neurogenesis

Fluoxetine (selective serotonin reuptake inhibitor)	Rats	Amygdala neuroplasticity, alleviates upregulation of synaptosomal polysialic neural cell adhesion molecule and reverses the inhibitory effects of chronic mild stress on spontaneous burst firing of medial prefrontal cortex pyramidal neurons

Tadalafil (phosphodiesterase inhibitor)	Rats	Suppresses maternal separation-induced apoptosis and increases cell proliferation in the dentate gyrus

Venlafaxine (serotonin/norepinephrine dual reuptake inhibitor)	Rats	Suppresses hippocampal apoptosis by upregulating brain-derived neurotrophic factor

Ketamine and lanicemine (NMDA receptor antagonists)	Rats	Increases mammalian target of rapamycin complex 1 (mTORC1) signaling by activating threonine kinase (AKT) and extracellular signal-regulated kinase (ERK) signaling pathways and increases synaptic number and function in the prefrontal cortex

Mecamylamine (nicotinic antagonist)	Rats	Increases PFC levels of BDNF and monoamines
